# When Falls Reveal More: The Geriatric Giant Unveils a Vestibular Schwannoma

**DOI:** 10.7759/cureus.85273

**Published:** 2025-06-03

**Authors:** Shivani Jani, George S Zacharia, Misbahuddin Khaja

**Affiliations:** 1 Internal Medicine, BronxCare Health System, New York, USA

**Keywords:** deafness, dizziness, falls, mri, schwannoma, vestibular

## Abstract

Vestibular schwannomas (VSs) are benign tumors originating from Schwann cells of the vestibular component of the eighth cranial nerve. They classically present as progressive unilateral sensorineural hearing loss, tinnitus, and imbalance, but the presentation in the elderly population can be atypical, resulting in diagnostic delays. Here, we describe a septuagenarian female who presented with recurrent dizziness and falls, diagnosed as VS, involving the left internal auditory canal. Neural and otogenic tumors are often overlooked causes of falls in the geriatric population, where dizziness and falls may be attributed to orthostatic hypotension, cardiovascular causes, or polypharmacy. However, early recognition is important, as timely neuroimaging helps with accurate diagnosis and management. MRI remains the gold standard modality. Management options range from observation to radiotherapy to surgical excision, with the choice depending on tumor size, patient comorbidities, and symptom severity. This case highlights the importance of heightened clinical suspicion and thorough evaluation, including early neuroimaging in elderly patients with recurrent falls and dizziness. A multidisciplinary approach involving neurology, neurosurgery, otolaryngology, and radiology ensures optimal diagnosis, treatment, and long-term follow-up.

## Introduction

Schwannomas are benign neoplasms of Schwann cells; the vestibular component of the eighth cranial nerve is one of the most frequent sites. Vestibular schwannoma (VS) accounts for approximately 8% of all intracranial tumors and 80-90% of cerebellopontine angle tumors [1,2]. The clinical presentation varies, with the most common symptoms being progressive unilateral sensorineural hearing loss, tinnitus, dizziness, and imbalance [2]. Diagnosis is often delayed in elderly patients because of the nonspecific nature of symptoms and overlapping etiologies, leading to multiple hospitalizations before definitive imaging and diagnosis.

Here, we present a case of a 77-year-old female with a left-sided VS who experienced persistent dizziness and recurrent falls, initially attributed to cardiovascular causes. A detailed workup ultimately led to the diagnosis, and she was managed non-surgically. This case highlights the diverse clinical presentations of VS, particularly in elderly patients, and emphasizes the importance of early diagnosis and intervention.

## Case presentation

A 78-year-old female with a medical history of hypertension, hyperlipidemia, hypothyroidism, and coronary artery disease, status post percutaneous coronary intervention with stent placement, presented to the ED after sustaining a fall. Antecedent to the fall, she experienced dizziness and a spinning sensation. Neither she nor her family members reported involuntary movements or loss of bowel or bladder control or loss of consciousness. She had no diarrhea, nausea, or vomiting, nor did she have any recent alteration of medications. She did not report any physical trauma, including head or scalp injuries.

The patient had a long-standing history of hearing difficulties attributed to presbycusis. Additionally, she reported multiple falls secondary to dizziness in the past, evaluated elsewhere; according to the patient, all previous workups were negative, and she had not responded to prescribed medications. She had undergone an extensive cardiac evaluation, including Holter monitoring, which yielded noncontributory findings in relation to her falls.

On presentation, her vital signs were stable, with no evidence of orthostatic hypotension. Neurological examination revealed no focal deficits except for reduced hearing, demonstrated by air conduction being greater than bone conduction on tuning fork testing. She exhibited no motor weakness or peripheral sensory loss. Cardiovascular, respiratory, and gastrointestinal examinations were unremarkable. The tympanic membranes were intact bilaterally, and the external auditory canals were patent. No visible injuries were noted, and there were no areas of bone tenderness or restricted joint mobility.

Labs were positive for iron deficiency anemia and indeterminate troponin levels (Table 1). The electrocardiogram was negative for any arrhythmia or acute ischemic changes. CT head without contrast was negative for any acute findings. Chest X-ray showed mild diffuse bilateral interstitial prominence, with new mild superimposed right basilar atelectasis. The patient was subsequently admitted to the telemetry unit due to persistent dizziness and a sensation of spinning.

**Table 1:** Summary of the hematology and biochemistry results

Parameter	Day 0	Day 2	Reference range
Hemoglobin	10.8	12.5	12.0-16.0 g/dL
Leukocyte count	5.8	8.3	4.8-10.8 k/μL
Platelets	199	233	150-400 k/μL
Sodium	136	139	135-145 mEq/L
Potassium	4.2	4.8	3.5-5.0 mEq/L
Blood urea nitrogen	27	20	6.0-20.0 mg/dL
Creatinine	1.2	0.9	0.5-1.0 mg/dL
Iron	6	-	65-175 ug/dL
Ferritin	16.4	-	13.0-150.0 ng/mL
Total iron-binding capacity	324	-	112-346 µg/dL
Calcium	8.4	9.3	8.5-10.5 mg/dL
Troponin	27	22	≤12 ng/L

**Figure 1:**
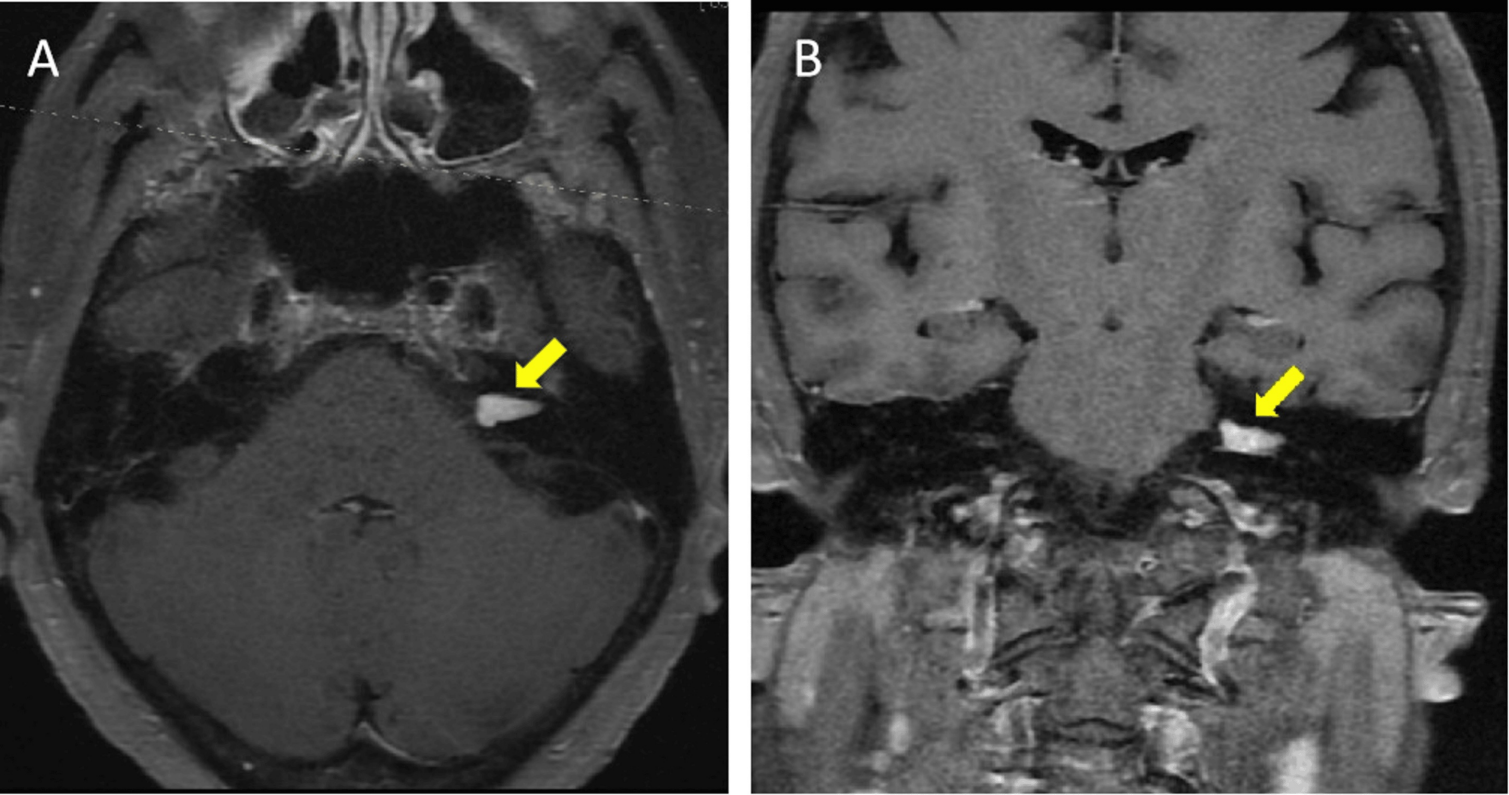
MRI brain T1 post-contrast series A 12 × 5 × 5 mm avidly enhancing mass filling the left internal auditory canal, extending slightly beyond the porus acusticus into the cerebello-pontine angle cistern. (A) Axial view. (B) Coronal view.

An echocardiogram reported an ejection fraction of 77.87% and a right ventricular systolic pressure of 41 mmHg. Given the patient’s persistent symptoms and negative cardiac workup, a contrast-enhanced MRI of the brain and internal auditory canal with contrast was performed to rule out neurological causes. The MRI revealed a left-sided schwannoma measuring up to 12 × 5 × 5 mm at the level of the internal auditory canal, extending slightly beyond the porus acusticus into the cerebellopontine angle cistern (Figure 1).

Otorhinology, neurosurgery, and neurology were consulted for opinions regarding further evaluation and management of the tumor. After a multidisciplinary discussion, the patient and the healthcare proxy decided to proceed with nonsurgical management, which included observation and serial imaging. Physiatry evaluation and counseling/support for fall risk reduction were provided; she was discharged on oral meclizine in addition to her regular medications for comorbid ailments.

## Discussion

Falls and instability are among the “geriatric giants,” a significant concern in the elderly population. Dizziness has multiple potential causes in the elderly, including cardiovascular and neurodegenerative diseases or medication side effects. This wide range of etiologies makes VS a less obvious differential diagnosis in an elderly patient with dizziness [3].

VSs are benign neoplasms of Schwann cells, primarily affecting the vestibular component of the eighth cranial nerve [1]. They account for approximately 8% of all intracranial tumors, with an incidence of 10.4 per million per year [4]. Schwannomas can be sporadic or can be seen as a part of neurofibromatosis type 2, which is an autosomal dominant disorder predisposing affected individuals to multiple CNS tumors, including bilateral VSs [5]. Neurofibromatosis type 2 results from an NF2 tumor suppressor gene mutation located on chromosome 22q [6].

VS usually presents with vertigo, tinnitus, and progressive unilateral sensorineural hearing loss. However, in elderly patients, symptoms such as dizziness, ataxia, and recurrent falls may predominate, leading to misdiagnosis. Larger tumors can also cause hydrocephalus and brainstem compression with symptoms such as facial paresthesia, vertigo, and headache [7].

VSs are often diagnosed due to acoustic symptoms such as hearing loss or vestibular symptoms like tinnitus or vertigo. Up to 20% of patients in otorhinolaryngology clinics have vestibulocochlear symptoms, where a lesion at the cerebellopontine angle is a differential diagnosis. Patients presenting with the aforementioned symptoms are usually investigated with otoscopy and pure tone audiometry, less frequently with MRI of the brain or internal acoustic meatus [8,9]. This may not always be the norm in elderly patients with comorbidities and/or background presbycusis presenting with isolated symptoms such as dizziness and falls. In such patients, investigations frequently focus on diagnosing cardiovascular etiologies, potentially leading to misdiagnosis or late diagnosis of the underlying schwannoma [3].

MRI is the preferred modality for diagnosing VS, while auditory brainstem response is a less preferred alternative. A systematic review performed by Fortnum et al. proved that there is minimal difference in sensitivity and specificity between gadolinium-enhanced T1-weighted MRI, which is regarded as the gold standard, and non-contrast T2-weighted scans. T2W sequences are also cost-effective and as efficient as available contrast imaging [10].

Management options for schwannoma include observation with serial imaging, radiotherapy, stereotactic radiosurgery (SRS), or surgical excision. SRS and fractionated radiotherapy have emerged as effective alternatives for small- to medium-sized VS. Studies have demonstrated that SRS offers excellent tumor control rates while minimizing complications compared to conventional surgery [11]. Radiotherapy is particularly beneficial for elderly patients and those with comorbidities who may not be optimal surgical candidates.

Surgical excision is the most commonly deployed treatment method, particularly for symptomatic or large tumors. Translabyrinthine, retrosigmoid, or middle fossa approaches could be selected based on tumor size, location, and the need for hearing preservation [12]. While surgery can effectively remove the tumor and alleviate symptoms, it carries risks such as facial nerve damage, cerebrospinal fluid leakage, and postoperative balance disturbances [7].

The prognosis for patients with VS is generally favorable. Long-term follow-up studies indicate that tumor control rates exceed 90% with either surgical or radiation therapy [13]. However, residual or recurrent tumors necessitate ongoing surveillance with serial imaging.

## Conclusions

VSs are a well-recognized cause of sensorineural hearing loss, dizziness, and imbalance. However, their presentation in elderly patients can be subtle and commonly mistaken for other medical conditions. This case depicts the diagnostic challenges and explains the importance of early neuroimaging in unexplained dizziness with recurrent falls. While surgical excision remains the cornerstone of treatment, radiotherapy offers an effective alternative for select patients. Multidisciplinary management involving otolaryngologists, neurologists, neurosurgeons, and radiation oncologists is essential for optimizing outcomes.
